# Pregnant women’s experiences of online prenatal education in Korea during COVID-19: a phenomenological study

**DOI:** 10.4069/whn.2024.09.09

**Published:** 2024-09-30

**Authors:** Hyun Kyoung Kim, Geum Hee Jeong, Hye Young Min

**Affiliations:** 1 Department of Nursing, Kongju National University, Gongju, Korea; 2 School of Nursing and Research Institute in Nursing Science, Hallym University, Chuncheon, Korea; 3 College of Nursing, Ewha Womans University, Seoul, Korea

**Keywords:** Digital technology, Distance education, Prenatal education, Pregnant women, Qualitative research

## Abstract

**Purpose:**

This study aimed to explore the significance and insights derived from the experiences of pregnant women in Korea who participated in online prenatal education during the COVID-19 pandemic.

**Methods:**

This study employed the hermeneutic phenomenology framework developed by Colaizzi. It involved 12 pregnant women who participated in online prenatal education provided by public health centers in Chuncheon, Korea. Data collection was achieved through in-depth interviews conducted in Korea from October 2021 to April 2022.

**Results:**

In total, 51 significant statements were extracted from the interview data and then categorized into 10 themes. Finally, three categories were formed by merging similar themes. The three basic categories of participants’ experiences of online prenatal education were “feeling of safety and comfort in body and mind,” “frustrated by a lack of interaction,” and “digital education being a double-edged sword.” Pregnant women expressed ambivalence regarding the benefits and drawbacks of the online educational experience. They desired more interactive and practical learning opportunities, even as they appreciated the comfort of learning remotely.

**Conclusion:**

This study revealed the phenomenon of online prenatal education as an advanced form of distance-based prenatal education instead of the traditional in-person classroom. To maximize the educational effectiveness of this new format, public health center policies must address the digital literacy gap and enhance accessibility by leveraging the immersive multimedia experiences that online education offers to pregnant women. We recommend that maternal healthcare providers adopt this innovative approach to prenatal education, utilizing distance education technology to improve participation and promote immersion.

## Introduction

Prenatal education is a learning process that equips pregnant women with the knowledge and skills needed for pregnancy, childbirth, postpartum health management, and newborn care. It serves as a vital intervention to enhance healthy childbirth outcomes and the physical and mental well-being of mothers postpartum [1]. Traditionally, prenatal education has been conducted in person at hospitals or health centers, often involving group sessions with pregnant women and their partners [2]. However, the onset of the coronavirus disease 2019 (COVID-19) pandemic led to restrictions on in-person group education programs for pregnant women due to infection risks. This prompted a shift in the education sector from traditional face-to-face interactions to remote learning methods [3]. The transition to non-face-to-face education required both educators and learners to adapt to new digital technologies and cope with reduced social support, a consequence of the lack of direct personal interaction [4].

In the broader context of educational transformation, prenatal education for pregnant women was either suspended or scaled back. Online prenatal education has emerged as a viable alternative in a society shifting away from face-to-face interactions, utilizing online platforms and video conferencing [3]. Although not previously the primary approach, online prenatal education interventions were explored even before the COVID-19 pandemic. These interventions typically involved web-based, mobile-based, social network service-based, and telephone-based methods reliant on internet connectivity [4]. The terminology for online education interventions varies, including terms like telehealth, mHealth, eHealth, distance education, video education, and digital education [5]. During the COVID-19 pandemic, there has been an increase in the activity of online education. More recently, innovative online education interventions employing virtual reality, augmented reality, games, simulations, the metaverse, and artificial intelligence have been initiated [6].

The topics covered in online prenatal education are similar to those in general prenatal education. It has been reported that pregnant women show a preference for education on newborn safety, delivery, and breastfeeding, in that order [7,8]. An integrative review study identified pregnancy management, fetal development, newborn safety, childbirth, and breastfeeding as the core topics of online prenatal education. Furthermore, a recent systematic review of randomized controlled trials, which examined online prenatal education as an intervention, found that the most commonly addressed topics were postpartum depression, anxiety, and breastfeeding. The primary outcomes measured in these trials included postpartum depression, anxiety, satisfaction, and mother-child attachment [4].

The effectiveness of online prenatal education has been reported to vary across studies. It was associated with a decrease in the risk of gestational diabetes, artificial abortion, postpartum infection, fetal distress, and neonatal malformation. Additionally, prenatal education focusing on mental health and nutrition during pregnancy was linked to a reduced risk of premature rupture of membranes and low birth weight [9]. An intervention that utilized an online prenatal education platform to provide peer support proved effective for breastfeeding [10]. Mobile application-based prenatal education interventions were shown to impact maternal parenting self-efficacy, prenatal attachment, social support, parental satisfaction, postpartum depression, and postpartum anxiety positively [11]. However, some studies found no significant effects of online prenatal education or no difference in outcomes compared to face-to-face education [12]. In terms of preferences among pregnant women, some studies indicated a preference for online education [13], while others reported increased stress, depression, rejection, anger, and lower parental attachment to online education, leading to a preference for face-to-face formats [14].

Over the past decade, there have been attempts to study online prenatal education interventions, but the benefits that pregnant women derive from such education have not been consistently reported [4]. Additionally, few studies have explored how pregnant women currently experience online education, especially in a post-pandemic society where non-face-to-face education has become the norm. It is crucial to vividly capture and understand the subjective experiences of pregnant women regarding online prenatal education. The shift to an online prenatal education system is not merely a temporary adjustment but part of broader macroscopic changes within the health system [2]. Therefore, it is necessary to examine the online prenatal education process as experienced by pregnant women and address any deficiencies to ensure educational quality. In this study, we aim to delve into the meaning and essence of the experiences of pregnant women who have engaged in online prenatal education, analyzing these experiences from their perspective using a phenomenological approach. Colaizzi’s phenomenological method is particularly well-suited for this research, as it aims to thoroughly understand the depth and complexity of personal experiences while ensuring that interpretations remain closely aligned with the unique narratives of pregnant women in the context of COVID-19. This study could provide vital insights for developing high-quality online prenatal education interventions, particularly when face-to-face education is not feasible.

This study aimed to clarify the meaning of the vivid experiences of pregnant women who participated in online prenatal education during the COVID-19 pandemic and to comprehend the essence of their experiences. The research question posed is, “What is the meaning of pregnant women’s experiences with online prenatal education?”

## Methods

Ethics statement: This study was approved by the Institutional Review Board of Hallym University (HIRB_IRB_2021-026). Participants were informed about the purpose and procedures of the study, and written consent was obtained. They were assured that their personal information would be kept strictly anonymous and confidential, with full protection of their privacy. Additionally, participants were informed that they could withdraw from the interview or study at any time.

### Study design

This study is a qualitative investigation that utilizes Colaizzi’s descriptive phenomenology research method [15] to explore the fundamental meaning of experiences associated with online prenatal education. It adheres to the Consolidated Criteria for Reporting Qualitative Research (COREQ) guidelines.

### Participant selection

The study participants consisted of pregnant women who enrolled in online prenatal education at the Chuncheon Public Health Center in Gangwon, Korea, from September 2021 to April 2022. The inclusion criteria were as follows: (1) pregnant women at any stage of pregnancy, as online prenatal education is typically offered from 4 to 38 gestational weeks, (2) women who exclusively participated in online prenatal education, and (3) women who were proficient in speaking, listening, and reading Korean. Women who attended any face-to-face sessions in addition to the online prenatal education were excluded from the study. We aimed to recruit at least 10 participants to ensure adequate data collection, and saturation was assessed to determine when no additional relevant information could be obtained [16].

Purposive sampling was conducted, and out of 32 pregnant women who had the opportunity to participate in the study, 12 were interviewed. By the time the 12th interview was completed, repetitive experiences had been noted, indicating that data saturation had been achieved, with no new concepts emerging.

### Data collection

Data collection was conducted by three researchers (HKK, GHJ, HYM) through one-on-one, in-depth interviews from October 7, 2021, to April 7, 2022. To recruit participants, we sought the cooperation of the head of the Chuncheon Public Health Center in Gangwon, Korea, specifically from the individual overseeing maternal and child health. Following the childbirth education sessions conducted via Zoom, the researcher provided details about the study and recruited participants. The prenatal education program was conducted through a series of videoconferences on the Zoom platform, totaling 8 hours over 4 weeks, with each session lasting 2 hours. These sessions included groups of 9 to 17 pregnant women. The weekly topics covered were: (1) health management during pregnancy, (2) pain management during childbirth, (3) prenatal environmental health practices (such as reducing exposure to environmental hormones, heavy metals, and micro-dust), and (4) postnatal care, which included breastfeeding and infant care. The program aimed to address pregnancy management, childbirth management, and postpartum care, incorporating lectures, explanations with YouTube and other audiovisual materials, and demonstrations using models of the breast, uterus, and baby. An employee at the Public Health Center’s Maternal and Child Center managed the technical aspects and monitored attendance.

The opportunity for participants to engage in interviews was presented at the conclusion of the final session. Within 1 week following the completion of prenatal education, individual phone calls were made to the pregnant women to confirm their interest and availability. For those interested in participating, consent forms and permission for recording were obtained via email or mobile phone, and an interview schedule was arranged. Only participants were present during the interviews.

For the in-depth interviews, participants were given the choice of time and mode. Due to COVID-19, none opted for face-to-face interviews. Four individuals participated in video interviews, while eight chose telephone interviews. Each session lasted between 40 minutes and 1 hour, with 1 to 2 interviews conducted per participant. The interviewers were the researchers themselves, who had prepared the main questions in prior meetings. The primary question was simplified for clarity: “What did your experience participating in online prenatal education during COVID-19 mean to you?” Additional exploratory questions included: “What are the positive and negative aspects of receiving education as a pregnant woman during the COVID-19 pandemic?”, “How does online prenatal education compare to face-to-face education?”, and “What improvements would you like to see in online prenatal education during COVID-19?” Following the interviews, participants received a mobile voucher worth 15 US dollars as a token of appreciation. The interview questions were refined iteratively throughout the data collection process to ensure they encompassed a wide range of experiences while allowing for a thorough exploration of each participant’s unique perspective. Participants were encouraged to thoroughly consider the questions and to listen attentively. Field notes were taken during the interviews, and the recorded data were manually transcribed using Microsoft Office 365 and Naver’s CLOVER automatic transcription programs.

### Data analysis

We used the philosophical basis and methods of Colaizzi’s descriptive phenomenology [15]. Colaizzi’s philosophy emphasizes immersing oneself in the event, vividly describing the subjective experiences of participants, and uncovering the core meaning of those experiences. His methodological approach involves extracting significant statements from the collected data, formulating meanings, and then categorizing these into thematic clusters by the researcher. This approach is characterized by its focus on the everyday experiences of participants, distinguishing it from other interpretive phenomenological methodologies [17]. Descriptive phenomenology offers a clear and systematic procedure for exploring experiences in fields where research is sparse [18]. It facilitates an understanding of the experiential process by identifying influencing factors and attributes. This is particularly useful in developing tools and constructing theories. Therefore, it is well-suited for this study, which aims to explore the essence of pregnant women’s experiences with online prenatal education during the COVID-19 pandemic.

The coded data were then shared and analyzed collaboratively through the Dropbox online platform. The coded files were examined during both video and face-to-face research meetings to discuss agreements and discrepancies in coding. We held over five research meetings, and for content with discrepancies, we initially listened to the explanations provided by the relevant researcher. If modifications were necessary, discussions continued until a consensus was reached on the new meaning assignment. In instances of persistent disagreement among researchers, the analyzed content was presented to the participants for confirmation, and the meaning was adjusted through a cyclical analysis method.

### Research rigor

The seven-step procedure used by Morrow et al. [18] for rigorous analysis is as follows: (1) To familiarize themselves with the data, the researchers read the participants’ statements multiple times. (2) To identify significant statements, they noted all sentences directly related to the phenomenon of pregnant women’s prenatal education experiences. (3) To create formulated meanings, the researchers carefully considered these significant statements with an open mind. (4) The researchers then derived themes by identifying common content among the formulated meanings and grouped these themes into clusters. (5) Once all themes were derived, the researchers comprehensively synthesized the information. (6) To generate the fundamental structure of the phenomenon, the researchers used concise wording to categorize and capture the essence of the phenomenon. (7) Feedback was obtained by asking the participants about their experiences to confirm the fundamental structure. The purpose of this respondent validation process was to ensure that the researchers maintained a phenomenological perspective based on the participants’ natural attitudes. A member validation procedure was also conducted among the researchers to determine whether the fundamental structure was reasonable and scientific. Additionally, to confirm the validity of the essential structure, opinions were sought from three professors specializing in women’s health nursing.

Creswell’s criteria [16] for securing qualitative validity in phenomenological research were applied. We prepared open-ended questions, modified them so that participants could express their experiences in their own words, and assisted participants in revealing their experiences in a relaxed atmosphere. We attempted to bracket our pre-understandings and explore the participants’ experiential world without preconceptions or biases. Our representative understanding was that online prenatal education has many advantages from the learner’s perspective, such as convenience, efficiency, accessibility, and satisfaction compared to face-to-face education, making it the most appropriate educational modality for pregnant women during the COVID-19 pandemic. We engaged each other in reflective thinking to recognize these preconceptions and not reflect or induce them in the participants’ interviews. To respect the unique life world of each participant, we attentively listened to their emotions and perceptions, repeatedly reviewing the transcripts and consulting field notes to immerse ourselves in their experiences and understand their perspectives. Each researcher independently coded the data using the Excel program. This process began with identifying significant statements, proceeded with deriving meanings, then organizing these into themes, and ultimately grouping them into broader categories.

For one participant who needed clarification on the meaning in the analyzed Excel coding table, we requested confirmation that the analysis accurately reflected the intended meaning. Additionally, to ensure the validity of our findings, we sought the opinions of three professors specializing in women’s health nursing, who were not part of the research team.

Furthermore, Creswell’s three criteria [16] for ensuring the reliability of phenomenological research were applied. First, the research procedure and questions were formalized into a protocol to enhance the reliability of the qualitative research. Second, we consistently documented the interview transcripts, field notes, and memos. Third, we independently conducted the coding work and regularly discussed the consistency of the coding in meetings.

### Researchers’ preparation

Researcher A and Researcher B are university professors with over 20 years and over 10 years of experience in qualitative research, respectively. Both hold doctoral degrees. Researcher C is a doctoral student with 5 years of experience in qualitative research. Throughout the 6-month interview period, the researchers maintained ongoing interactions and discussions to focus on the direction of the research.

### Relationship with participants

None of the researchers involved in the interviews had a personal relationship with the participants. The participants were aware that the researchers included university professors and a doctoral student.

## Results

### Characteristics of the participants

The 12 participants ranged in age from 24 to 42 years, with gestational ages spanning 12 to 37 weeks. Their number of pregnancies varied from 1 to 5, while the number of deliveries ranged from 0 to 1, and the number of children also ranged from 0 to 1. The participants’ occupations were diverse, including five homemakers, two freelancers, two teachers, one instructor, one banker, and one officer. Educational backgrounds varied as well: 10 participants had completed college, one had finished high school, and one had a graduate degree. Health issues reported by the participants included back pain, pelvic pain, vaginal spotting, morning sickness, cystitis, and a history of infertility treatment (Table 1).

### Participants’ experiences of online prenatal education

A total of 51 significant statements were extracted from the interview data and subsequently categorized into 10 themes. After further discussion, these themes were consolidated into three categories (Table 2).

### Category 1: Feeling of safety and comfort in body and mind

Participants reported an increased sense of safety and comfort during online prenatal education, attributed to the reduced risk of infection, the convenience of learning from home, the ability to multitask, the involvement of family members, and decreased requirements for preparation and childcare. This approach also resulted in time savings and reduced transportation costs.

#### Education in a “quarantine-controlled” situation

During the COVID-19 pandemic, a period marked by stringent quarantine measures and a focus on social distancing and infection prevention, online prenatal education emerged as the safest option for pregnant women and their babies, who are particularly vulnerable to infections.

“Baby fairs are being held now, but should I go at this time (COVID-19)? So I’m so worried about getting infected that I’m not going... It’s difficult to go anywhere due to the coronavirus situation... I was glad that both my baby and I learned safely in a comfortable place. Online education had the best accessibility.” (Participant 3)

“I thought the education was pretty good. Considering there was COVID-19, and pregnant women are a high-risk group, I liked that it was done online.” (Participant 6)

#### Comfortable with fewer constraints

Even during the challenging periods of pregnancy when movement was difficult, participants felt comfortable attending sessions without any limitations. They had the opportunity to engage in educational activities alongside their husbands and could participate in these sessions while working or engaging in other activities, all without feeling any stress.

“I had preterm labor… my body was a bit uncomfortable… It was less tiring. It wasn’t going out, and I usually listened while lying on my side on the sofa. So there was less tension, and I could listen comfortably. Since I was alone during the day, it was an excellent time to listen to the education and rest… I could participate while doing anything, so there was no burden in terms of time or transportation.” (Participant 9)

“I was feeling a bit uncomfortable due to the pregnancy, so it was nice to be able to attend comfortably.” (Participant 6)

#### Home-based cozy environment

Participants appreciated the flexibility of joining sessions from the comfort of their homes or any cozy location, where they could lie down, sit, or move around freely. They also valued the option to turn off their cameras if they wished.

“I would find a quiet place in my room and sit or lie down comfortably to listen so there was no noise, and I could concentrate better. Since I wanted to listen anyway, it felt like studying at home, so I could immerse myself more and be satisfied.” (Participant 11)

“First of all, not having to go in person was definitely an advantage. During the four sessions of the course, I was experiencing severe morning sickness. If the sessions had been held offline, there might have been a couple of times when I wouldn’t have been able to attend.” (Participant 5)

#### No makeup, no dress-up

They found joining online sessions convenient as it eliminated the need for makeup, dressing up, or preparing to go out. Additionally, they avoided the need for arranging babysitting and also saved on transportation costs.

“If I have to go out, I have to change my dress and makeup… and take a car and buy a drink. It may be challenging to go out. Especially in Chuncheon, it isn’t easy to use public transportation, and the intervals between buses are also long.” (Participant 6)

### Category 2. Frustrated by a lack of interaction

Participants expressed frustration with the limited interaction in online prenatal education, highlighting issues such as one-way lectures, insufficient feedback, and a lack of connection. The social isolation and absence of physical presence further contributed to feelings of loneliness and made them hesitant to ask questions.

#### One-way lectures

Participants found online lectures lacking in direct face-to-face interaction, meaning that they received minimal or no responses to their questions and unsatisfactory feedback from instructors. Additionally, turning off their cameras hindered the ability to establish eye contact and connection.

“Oh, during the lecture, I asked the same question in the 1st and 2nd sessions, but I didn’t get the feedback I wanted, so I stopped asking questions. Also, it’s not about looking at each other and asking questions.” (Participant 4)

“I don’t know if it’s because I didn’t turn on the camera, but if I looked at the camera directly, I could have had eye contact and rapport with the teacher. Not so much when I turned off the camera. Isn’t this the limitation of online education?” (Participant 6)

#### Loneliness due to social isolation

Participants were eager to share interests and engage in discussions with other women participating in the education program, but found it difficult to communicate and establish connections, which led to feelings of isolation. Although they did not consider online education to be inaccessible, they found communicating through a small phone screen to be challenging.

“I don’t know if I’m not sociable or if it’s awkward because it’s online, but I want to get closer to pregnant women at the same number of weeks, but it was difficult. There were also some parts that made me cautious.” (Participant 6)

“First, non-face-to-face education is so common now that I didn’t think the accessibility was terrible. Still, it’s difficult to watch on a cell phone because the screen is small, so in my case, it was convenient to watch on a computer, but that made it difficult to communicate” (Participant 8)

#### No questions due to others’ gaze

Participants had personal questions they wanted to ask but were embarrassed about what others might think, hesitating for fear of delaying the session. This hesitation resulted in their passive participation. Although they felt comfortable with their cameras off and remaining unseen, they also felt pressured by the possibility of the instructor asking them to turn their cameras on.

“Instead, it was more challenging to ask questions. I also thought, ‘What if I ask a question about this lecture now, and it takes longer?’ I had a more profound feeling of ‘I’ll just have to find out myself’ rather than face-to-face. It’s not necessarily because of time, but when you do it online, you tend to approach lectures passively. Of course, I asked a few questions during the middle of the question session, but.” (Participant 4)

“The screen is not visible, so it’s okay to hear the voice, but if you ask me to show my face, it would be very burdensome because I’m usually on leave at home and listening comfortably” (Participant 7)

“The online classes were a bit challenging. I felt embarrassed to ask personal questions, and I was also concerned that it might delay the class.” (Participant 6)

#### Category 3. Digital education being a double-edged sword

Digital education in prenatal learning provides benefits such as enhanced visual engagement and creative stimulation. However, it also presents challenges including passive learning, diminished concentration, difficulties in memory retention, and heightened digital inequality. This inequality is particularly intense in rural areas where access to technology is often limited.

#### More than words

Visual learning via videos on phones and computers enhanced their understanding and engagement with the educational content. Education that incorporated exciting music and art stimulated their imagination.

“Online education is so common now that it’s a trend. It will develop more in the future. If you have virtual reality and music, you can enjoy learning. It would be nice to have more opportunities like this. I don’t know much about information and communication, but I’ve improved dramatically while receiving education.” (Participant 10)

#### Relaxation but not concentration

Although participants felt comfortable with their anonymity, it resulted in passive involvement and diminished engagement. The sessions employed a rapid-fire format, which subsequently made it challenging for attendees to remember the details clearly.

“I felt comfortable, but sometimes I just stared blankly at the screen... it helped a lot, and first of all, I learned things I didn’t know, but the regrettable thing was that there was no experience, so I just listened and passed by. The lecture materials were sent, so I reviewed them, but even if I reviewed them all, I didn’t feel the vividness of that time. I tried to take notes separately while listening, which helped a lot then. Since they tell you a lot at once, I’ll forget when I turn around (laughter). I’ll have to take [the materials] out again someday, and that’s regrettable.” (Participant 8)

#### Marginalization due to digital inequality

Participants experienced digital inequality in online education access and quality due to varying internet and electronic device capabilities.

“The internet didn’t work, so I was supposed to go a little earlier, but I got cystitis, so the online education period overlapped. Going to the hospital in the countryside was difficult, because the Wi-Fi didn’t work well, (um) so I was at my country house and could only listen once.” (Participant 7)

## Discussion

This study utilized Colaizzi’s descriptive phenomenological method [15] to conduct in-depth interviews with 12 pregnant women, aiming to elucidate the profound experiences of those who participated in online prenatal education. The analysis yielded 10 themes and three categories. These categories included “feeling of safety and comfort in body and mind,” “frustrated by a lack of interaction,” and “digital education being a double-edged sword.” The core essence of the online prenatal education experience was explored through phenomenological writing, guided by the identified theme clusters.

The first category—“feeling of safety in body and mind”—refers to the experience of safely accessing education during the pandemic through online prenatal classes. This method allowed participants to alleviate the stress of the situation while continuing their education. Even before the pandemic, attempts were made to provide education in a “quarantine-controlled” environment via a webpage dedicated to breastfeeding [19], which proved to be a recommendable approach for midwives as it effectively conveyed practical and evidence-based knowledge. Online prenatal education, in particular, was beneficial for the mental health of pregnant women, as it helped reduce feelings of sadness, depression, and stress, while offering mental support [20]. During the pandemic, pregnant women experienced greater fear and anxiety about childbirth compared to their non-pregnant counterparts; however, online prenatal education helped alleviate these concerns [21]. The convenience of online education allowed pregnant women to learn from the comfort of their homes, which added to their comfort by enabling them to adopt various relaxed postures, such as lying down or sitting on a sofa, without the need to maintain a specific demeanor [2]. Additionally, it eliminated the need for public transportation to attend classes, as well as the requirement to wear makeup or change clothes, thus providing economic and logistical advantages [2].

The second category, “frustrated by a lack of interaction,” describes the negative aspects of distance education, where the desire for closeness with other pregnant women goes unmet, communication between educators and learners is disrupted, and both social support and social communication are significantly reduced [4]. Results indicate that while online breastfeeding education enhances knowledge and skills, it does not improve co-parenting relationships, highlighting the challenges online education faces in facilitating effective communication between instructors and learners, as well as among the learners themselves [22]. However, integrating two telephone interventions into prenatal education via a mobile application has shown positive effects on social support [23]. Therefore, to enhance social communication in online education, it is essential to employ strategies such as using social network services, forming peer groups, providing additional one-on-one time with educators, and incorporating face-to-face interactions [24]. This study reveals that participants experienced negative feelings, such as loneliness during lectures with minimal interaction and hesitation to ask questions due to the presence of unfamiliar individuals. These feelings stemmed from a lack of opportunities to connect with other pregnant women, underscoring the need for stronger social support in future online educational settings.

“Digital education being a double-edged sword” refers to the experience of accessing multimedia advantages such as videos, photos, and music more vividly through digital technology, which is generally satisfactory. However, it also highlights the lack of human interaction and the inequality in accessibility caused by the digital divide. Participants noted that learning with audiovisual effects goes beyond mere words, showing a preference for the accessibility of digital technology in online education over traditional face-to-face settings. Most experts in women’s health are satisfied with online education, finding it safe, effective, learner-centered, and timely. The learning retention rate was reported to be 63.4%, indicating high-quality outcomes [25]. From the learner’s perspective, there are concerns that online education may reduce concentration due to the more relaxed posture compared to traditional settings. This suggests a lack of intangible factors such as the atmosphere, tension, and enthusiasm typically found in face-to-face education. Globally, Internet and public Wi-Fi penetration rates vary significantly by region and country, leading to potential alienation due to another form of digital divide. One participant expressed regret that she could not access education after moving to a rural area without an internet connection, highlighting how technological gaps can widen health disparities. Therefore, ensuring equal access to online education is crucial [26]. While online education offers numerous benefits for the digital generation, it has also been identified as a factor that exacerbates health inequalities post-pandemic, influenced by race, region, economy, and education [24].

The strength of this study lies in its timing and methodology. It is a qualitative study conducted during a period of heightened quarantine measures, at the peak of the pandemic, when in-person prenatal education was suspended. This study explores the vivid experiences of pregnant women engaging in online prenatal education, a new approach at the time. By using in-depth interviews, it identifies the advantages and disadvantages of remote learning. Thus, this research aids in determining the contextual superiority of face-to-face versus remote education. This study offers valuable insights for public health centers and hospitals looking to implement prenatal education online. It helps them to mitigate the disadvantages and enhance the benefits of online learning. Furthermore, this research contributes to the foundational studies that inform government policies on online prenatal education. These policies aim to maximize educational outcomes and improve accessibility, thereby addressing economic disparities and bridging digital literacy gaps through the enriched multimedia experiences that pregnant women encounter in online education.

This study has several limitations that should be considered when interpreting the findings. It was conducted during the COVID-19 pandemic, a time when face-to-face prenatal education options were unavailable to the participants. Consequently, their experiences with prenatal education were likely limited primarily to online formats. This restriction may have shaped the participants’ perceptions and feedback, as they lacked a direct comparison with traditional face-to-face education during this period. Additionally, due to pandemic-related restrictions, the interviews were conducted via Zoom meetings and phone calls. This method may have diminished the ability to detect non-verbal cues and expressions, potentially affecting the depth of the data collected. These factors should be taken into account when applying the findings to other contexts or populations.

The findings of this study suggest that it would be worthwhile to compare the effects of non-face-to-face and face-to-face prenatal education in the post-pandemic era. Additionally, it recommends conducting an experimental study to assess the effectiveness of an exchange program among pregnant women, which would incorporate social interaction into online prenatal education. It is also suggested that a hybrid model of prenatal education, combining online and face-to-face elements, be developed and implemented. This approach aims to mitigate the disadvantages of online education identified in this study while maximizing its advantages.

## Figures and Tables

**Table 1.** Participants’ characteristics (n=12)

No.	Age (year)	Gestation age (week)	Gravidity	Parity	Number of children	Education level	Job	Health problems
1	40	24	5	0	0	College	Housewife	Infertility treatment
2	38	12	1	0	0	College	Teacher	None
3	35	36	3	1	1	College	Freelancer	Vaginal spotting
4	40	20	1	0	0	College	Officer	None
5	41	26	1	0	0	College	Teacher	Morning sickness
6	35	37	1	0	0	High school	Housewife	Cystitis
7	42	20	1	0	0	College	Banker	Husband’s infertility treatment
8	34	20	1	0	0	College	Freelancer	None
9	35	20	2	1	1	College	Lecture	None
10	24	24	1	0	0	College	Housewife	Pelvic pain
11	32	27	1	0	0	College	Housewife	None
12	35	30	1	0	0	Graduate school	Housewife	Back pain

**Table 2.** Categories and themes of participants’ experience of online-based prenatal education

Categories	Themes	Significant statements
Feeling of safety and comfort in body and mind	Education in “Quarantine-controlled” situation	• The best way to choose
		• A safe way for me and my baby
		• Social distancing
		• Fear of infection
		• Vulnerability
		• Quarantine policy
	Comfortable with fewer constraints	• Listening to lectures while doing other activities
		• Participating with family
		• Selective listening
		• No tension
	Home-based cozy environment	• Free postures
		• Comfortable body
		• Off the camera
		• Freedom of movement
	No makeup, no dress-up	• No need to get ready to go out
		• No need to dress up or put on makeup
		• No babysitting
		• Saving transportation expenses
Frustrated by a lack of interaction	One-way lectures	• Lack of personalized attention
		• No responsiveness
		• Unsatisfactory feedback
		• Indirect interaction
		• No eye contact
		• Lack of sense of connection
	Loneliness due to social isolation	• Lack of intimacy
		• Need for social interaction
		• Barrier to connecting with others
		• Feeling of isolation
		• Non-face-to-face communication
	No questions due to others’ gaze	• Passive participation
		• Hesitation
		• Shyness
		• Online privacy
		• Lack of physical presence
		• Fear of pressure
Digital education being a double-edged sword	More than words	• Expanding beyond tradition
		• Visual learning
		• Clear understanding
		• Deep engagement
		• Image inspiring
		• Exciting music and art
	Relaxation but not concentration	• Anonymity and comfort
		• Passive learning
		• Lack of immersion
		• Inability to capture liveliness
		• Rapid-fire format
		• Memory retention challenge
	Marginalization due to digital inequality	• Digital divide in a rural setting
		• Accessibility challenge
		• Technological infrastructure
		• Barriers to accessing a digital resource
		
